# Properties That Potentially Limit High-Level Blends of Biomass-Based Diesel Fuel

**DOI:** 10.1021/acs.energyfuels.4c00912

**Published:** 2024-05-07

**Authors:** Robert L. McCormick, Gina M. Fioroni, Nimal Naser, Jon Luecke

**Affiliations:** National Renewable Energy Laboratory, 15301 Denver West Parkway, Golden, Colorado 80401, United States

## Abstract

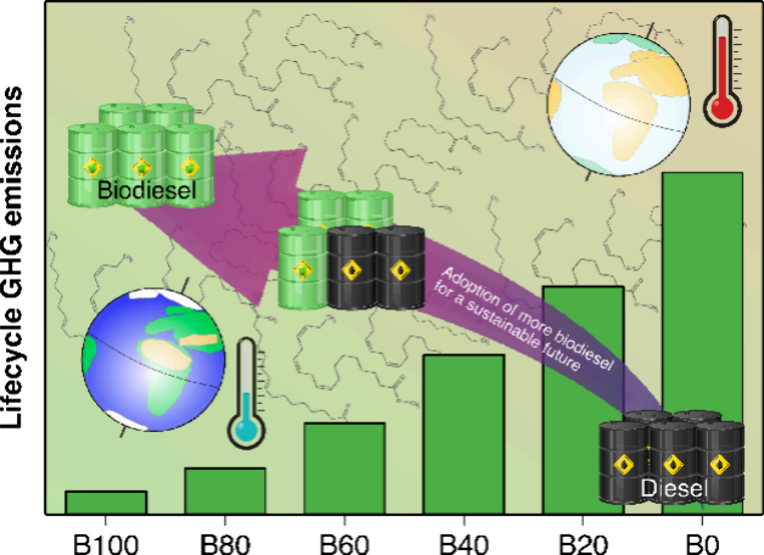

While today’s biomass-based diesel fuels are used at relatively low blend levels in petroleum diesel, decarbonization of the heavy-duty trucking and off-road sectors is driving increasing use of higher level blends and the combination of hydroprocessing-derived renewable diesel (RD) with biodiesel (fatty acid methyl esters) to create a 100% renewable fuel. However, little data are available on the properties of biodiesel blends over 20 vol % into RD or conventional diesel, despite the potential for properties to fall well outside the normal range for diesel fuels. Here, we evaluate the properties of 20–80% blends of a soy-derived biodiesel into RD and petroleum diesel. Properties measured were flash point, cloud point, cetane number, surface tension, density, kinematic viscosity, distillation curve, lower heating value, water content, water solubility in the fuel, lubricity, and oxidation stability. Density and viscosity were measured over a wide temperature range. A key objective was to reveal properties that might limit blending of biodiesel and any differences between biodiesel blends into RD versus petroleum diesel and to understand research needed to advance the use of high-level blends and 100% renewable fuel. Properties that may limit blending include the cloud point, viscosity, distillation curve, and oxidation stability. Meeting cloud point requirements can be an issue for all distillate fuels. For biodiesel, reducing the blend level and use of lower cloud point hydrocarbon blendstocks, such as No. 1 diesel or kerosene, can be used in winter months. Alternatively, a heated fuel system that allows for starting the vehicle on conventional diesel before switching to pure biodiesel (B100) or a high-level blend has been successfully demonstrated in the literature. Some biodiesels can have kinematic viscosity above the upper limit for diesel fuels (4.1 mm^2^/s), which will limit the amount that can be blended. Biodiesel boils in a narrow range at the very high end of the No. 2 diesel range. Additional research is needed to understand how the high T90 of B100 and high-level blends and the very low distillation range of B100, some RD samples, and high-level biodiesel blends impact lube oil dilution, engine deposits, and diesel oxidation catalyst light-off. Blending with No. 1 diesel or kerosene or biodiesel-specific engine calibrations may mitigate these issues. Oxidation stability of higher level blends is poorly understood but may be addressed through the increased use of antioxidant additives. Finally, high-level biodiesel blends and B100 will have significantly higher density, viscosity, and surface tension compared to conventional diesel. In combination with the high boiling point, these properties may impact fuel spray atomization and evaporation, and additional research is needed in this area.

## 1 Introduction

Transportation is currently the largest greenhouse-gas-emitting sector of the United States energy economy^1^ and the third largest sector globally.^2^ While small- and medium-sized vehicles may ultimately be electrified and decarbonized using green electricity, larger vehicles, such as heavy-duty long-haul trucks, large off-road equipment, marine ships, and transcontinental/intercontinental aircraft, will likely be powered by low-carbon-intensity liquid fuels. For example, a recent analysis of decarbonization of on-road medium- and heavy-duty vehicles found that, under a set of favorable but reasonable assumptions for the introduction of battery and fuel cell technologies, internal-combustion-engine-powered vehicles would represent 20% of the vehicle stock in 2050 but consume over 50% of the energy used by this sector.^3^ Clearly, large volumes of low-carbon liquid fuels will be required, and these must be available and compatible with vehicles in neat form, without blending with petroleum-derived fuel.

The two low-net-carbon biomass-based diesel fuels available today are biodiesel and hydrocarbon renewable diesel (RD), both produced from fats, oils, and greases. Biodiesel consists of fatty acid methyl esters (FAMEs) and is produced by the transesterification of fats, oils, and greases with methanol. RD is produced by hydroprocessing and isomerization of the same feedstocks. These fuels can both achieve life cycle greenhouse gas emission reductions of 40–86% relative to petroleum diesel, depending upon feedstock and assumptions regarding land use change.^4^ In 2022, the consumption of biodiesel and RD in the United States was 11.8 billion L, of which 6.1 billion L (1.6 billion gallons) was biodiesel.^5^ For the European Union in 2022, combined biodiesel and RD use was 16.1 billion L, of which 12.1 billion L (3.2 billion gallons) was biodiesel.^6^

Today in the United States, biodiesel and RD are primarily used as relatively low-level blends in petroleum diesel to meet renewable fuel standard regulatory requirements. An exception is California, where blends of biodiesel into RD to create a 100% low-carbon fuel have begun to be used to meet state-specific regulatory requirements (California Low Carbon Fuel Standard) as well as evaluated for reducing tailpipe emissions.^7^ The motivation for blending biodiesel into RD is that, for standalone production facilities, biodiesel is significantly less expensive to produce.^8^ Much of the production cost difference disappears if the RD is produced in a petroleum refinery. Biodiesel also reduces engine-out particulate matter mass emissions and produces more reactive particles.^9−11^ This causes a reduction in diesel particle filter regeneration events and lower temperature regeneration, leading to reduced fuel consumption. Blends containing 20 vol % or higher levels of biodiesel in either conventional diesel or RD will be increasingly important across the United States and globally for meeting heavy-duty transport decarbonization goals, along with other forms of low-net-carbon diesel yet to be commercialized.^12^

Soy oil, yellow grease, and corn oil were the most common feedstocks used for biodiesel production from 2022 to 2023 in the U.S.^13^ Extensive property data are available for soy biodiesel blends up to 20 vol % in North American conventional diesel,^14−16^ but there is little data on higher blends. Of the few published studies, Yoon and colleagues measured density and viscosity for soy biodiesel blends up to 100% into a petroleum diesel over a broad temperature range.^17^ Generally, the density and viscosity increased with biodiesel blending but remained in the normal range for diesel fuels. Candeia et al. presented property results for 5, 15, 25, and 50 vol % blends of soy biodiesel into conventional diesel.^18^ They observed reduced volatility (increased distillation T50) and increased viscosity with increasing biodiesel blend levels. Luning Prak et al. published extensive data on biodiesel blends up to 100%, including from soy and corn oils, in military jet fuel, JP-5.^19^ Density, kinematic viscosity, flashpoint, surface tension, and bulk modulus all increased with biodiesel blending. In contrast, multiple studies on the properties of biodiesel made from tropical oils blended into petroleum diesel have appeared.^20−23^ These include biodiesel from *Jatropha*, *Moringa*, palm, and coconut, among others. As observed for soy biodiesel, density, kinematic viscosity, and flashpoint increase, while oxidation stability can decrease, with biodiesel blending. As revealed in these studies, conventional diesel fuel from tropical areas tends to have a much higher cloud point than is typical in the U.S. and Europe, in the range of 8–12 °C, and thus, impacts on cold temperature properties are different.

In contrast, almost no information about biodiesel blended into RD has been published. A detailed composition and property assessment for multiple samples of RD has been presented,^24^ and Lapuerta et al. reported properties of a limited range of ternary blends of biodiesel, RD, and petroleum diesel.^25^ However, there is not a significant database or detailed understanding of the properties of biodiesel blends with RD nor high-level blends with conventional diesel, including a discussion of factors that may limit blending.

Because of the specific chemistry and properties of biodiesel, there may be several issues that arise as blend levels increase. Meeting wintertime cloud point requirements is a challenge for all distillate fuel producers, including petroleum refiners. Biodiesel tends to have a cloud point of roughly 0 °C or higher, making the use of high-level blends or pure biodiesel (B100) challenging in much of the world during the winter. Biodiesel has a slightly lower energy content per mass or volume than conventional diesel, requiring higher fueling rates to produce a given engine load. Engine control systems not designed for biodiesel blends may misinterpret this fueling demand and operate the engine at suboptimal settings.^26^ Biodiesel boils above the allowable T90 maximum for conventional diesel (338 °C in ASTM D975). As more biodiesel is blended, distillation T90 will increase, potentially to levels that cannot be measured using atmospheric distillation or that cause engine operating problems, such as high lube oil dilution or engine cylinder deposits. Oxidation stability may also be reduced as more biodiesel is blended, requiring higher levels of antioxidant additives. Properties that affect spray atomization, boiling point, density, viscosity, and surface tension, will typically increase in value with biodiesel blending, reducing spray atomization quality^27,28^ and potentially leading to engine performance and emission impacts.

Thus, there is a significant gap in our knowledge of fuel properties of biodiesel blended into both conventional diesel and RD and a poor understanding of how fuel properties could limit blending based on engine performance problems. We examine blends at 20, 40, 60, 80, and in some cases 90 vol % of biodiesel produced from the most common feedstock used in the United States, soybean oil, with conventional diesel and RD. For some properties, more than one conventional diesel or RD is used for blending. The neat blend components are also characterized. Fuel evaluations go well beyond ASTM standard requirements to include viscosity and density over a wide temperature range, surface tension, distillation, including atmospheric, vacuum, and gas chromatography (GC) simulation, and oxidation stability by Rancimat and PetroOxy induction times, among other properties. How certain properties might be problematic for engine operation and, therefore, limit blending is discussed in light of the research needed to advance the use of high-level and 100% low-carbon fuels.

## 2 Materials and Methods

Conventional petroleum diesels [ultralow-sulfur diesel (ULSD)], hydrogenated ester and fatty acid RD, and soy biodiesel were obtained from commercial suppliers. The biodiesel is of 1B S15 grade (defined by ASTM D6751), the most common grade used in the United States. Biodiesel from more saturated feedstocks, such as beef tallow or palm oil, will have a higher cloud point, viscosity, and cetane number (CN).^29^

Blends of the biodiesel into either ULSD or RD were prepared gravimetrically using the known fuel densities to target specific volume percent blending levels. This was done to be consistent with the market and ASTM specification practice to report oxygenate blending as volume percent. The lower heating value of the blends was calculated using the blend component values and mass percent. ASTM and EN test methods used to measure blend fuel properties are shown in Table 1. Instrumentation used to perform these methods is listed in Table S-1 of the Supporting Information, along with the volume of fuel required. ASTM or EN method repeatability (95% confidence interval for measurements made in the same lab by the same operator) is reported with the results. All fuels were stored in sealed steel cans in a refrigerator to prevent oxidation and ingress of water during the study.

**Table 1:** Blend Property Measurement Methods

property	method	comments
kinematic viscosity (mm^2^/s)	D7042	viscosity temperature sweeps
density (g/mL)	D7042	density temperature sweeps
surface tension (mN/m)	D1331	Wilhelmy plate method
flash point (°C)	D6450	continuous closed cup method
cloud point/freeze point (°C)	D5773/D5972	PhaseTech method
distillation T90 (°C)	D86	atmospheric pressure
distillation T90 (°C)	D1160	vacuum distillation
distillation T90 (°C)	D2887	simulated distillation (GC) procedure B, D86 correlation
CN	D8183	indicated cetane number (ICN)
lower heating value (MJ/kg)	calculated (ULSD, RD, and B100 by D240)	calculated from blend component values and blend composition
water content and saturation water solubility (ppm)	D6304	Karl Fischer titration
lubricity (μm)	D6079	HFRR method
oxidation stability (h)	EN 15751	Rancimat induction time
oxidation stability (min)	D7545	PetroOxy induction time

In describing the results, we refer to several ASTM standards, which apply in the United States and many other countries. Conventional diesel and hydrocarbon RD are required to meet the requirements of ASTM D975 Standard Specification for Diesel Fuel, which also apply to biodiesel blends up to 5 vol %. B100 biodiesel is required to meet ASTM D6751 Standard Specification for Biodiesel Fuel Blendstock (B100) for Middle Distillate Fuels, and blends of biodiesel from 6 up to 20 vol % into hydrocarbon fuels (whether conventional or renewable) are required to meet ASTM D7467 Standard Specification for Diesel Fuel Oil, Biodiesel Blend (B6 to B20). There are currently no standard requirements for biodiesel blends above 20 vol %, and the current data set is intended to inform ongoing deliberations about what high blend standards should look like. While not used in the U.S., EN 15940 Automotive Fuels—Paraffinic Diesel Fuel from Synthesis or Hydrotreatment—Requirements and Test Methods applies to RD blends and blends of RD with biodiesel up to 7 vol%.

## 3 Results

### 3.1 Blend Component Properties

Characterization data for the main fuels used for blending in this study are given in Table 2. RD-1 was primarily used for blending, but properties of two additional RD (referred to as RD-D and RD-G) from different producers are reported in Table S-2 of the Supporting Information; these data and results by Smagala et al.^24^ suggest that RD fuels have a relatively narrow range of properties. The RD fuels were all compliant with D975 and EN 15940 for the properties measured. ULSD-A was the primary diesel used for blending with biodiesel and has a relatively high T90, making it potentially the worst case for the study of high-level blend impacts on this parameter. Three other ULSD samples were also used for specific properties and are described in Table S-3 of the Supporting Information. ULSD-C was used for cloud point measurements and was prepared by blending ULSD-A with another diesel with a cloud point of −5.3 °C (ULSD-B) to obtain fuel with a cloud point similar to that of RD-1. ULSD-D was used for comparison in oxidation stability measurements because of its relatively short PetroOxy induction time of 55 min.

**Table 2:** Properties of Neat Fuel Blend Components

property	method	RD-1	biodiesel	ULSD-A
flash point (°C)	D6450	72	161	73
cloud point (°C)	D5773	–19.6	–0.6	–26.2
distillation T90 (°C)	D86	296	352^a^	333.9
kinematic viscosity at 40 °C (mm^2^/s)	D7042	3.062	4.055	3.239
density at 15 °C (g/mL)	D7042	0.780	0.884	0.843
surface tension at 20 °C (mN/m)	D1331	26.1	30.6	27.9
water content (ppm)^b^	D6304	16/85	193/1567	26/80
CN (as ICN)	D8183	79	52	48.7
sulfur (ppm)	D5453	<0.5	<0.5	5.3
hydrogen (wt %)	D5291	14.824	11.457	13.34
lower heating value (MJ/kg)	D240	43.91	37.33	43.12
oxidation stability (h)	EN 15751		4.7	
oxidation stability (min)	D7545	93	19	79
lubricity (μm)	D6079	410,^c^ 590,^d^ and 350^e^		390
aromatics (vol %)	GC × GC			27.8
monoglycerides (wt %)	D6584		0.113	
cold soak filterability (s)	D7501		85	

a D1160.

b As received/saturation.

c As received.

d After treating with silica gel to remove the lubricity additive.

e After silica gel treating and blending with 5% biodiesel.

Biodiesel and RD generally have lower sulfur contents than ULSD, and the samples described in Table 2 had sulfur below detection on the method used. The mean sulfur content of biodiesel in the United States is about 4 ppm.^30^ B100 has properties that are typical of soy biodiesel in terms of cloud point, kinematic viscosity, density, and cetane number. Oxidation stability as the Rancimat induction period (EN 15751) was 4.7 h, above the minimum requirement of 3 h in D6751. This value, while meeting the specification, is well below the U.S. market average value of over 9 h.^30^ As shown below, B20 blends met the 6 h minimum requirement in D7467; however, the relatively low induction period value presents a worst case for blending at levels above B20.

RD-1 as received had a lubricity value of 410 μm (wear scar diameter), well below the maximum allowable value of 520 μm in D975. Given that relatively pure hydrocarbons, including ULSD, typically have poor lubricity, this strongly suggests that RD-1 had been treated with a lubricity improver additive. Several studies have shown that blending biodiesel into ULSD or highly paraffinic Fischer–Tropsch diesel can obviate the need for lubricity additives.^31^ Lubricity additives are typically carboxylic acids,^32^ and these were removed from RD-1 by mixing with activated silica gel, resulting in a wear scar of 590 μm, a failing value. Blending of 5% biodiesel into this fuel imparted more than adequate lubricity to the RD, with a wear scar of 350 μm. Note that the as-received RD-1, with a lubricity additive, was used for preparing biodiesel blends and measurement of other properties.

While fuels as produced contain little to no water, in-use they typically contain low levels of dissolved water that are picked up in the fuel distribution system. In many cases, retail and even terminal fuel tanks will have a water layer in the bottom of the tank such that the fuel is saturated with water.^33^ The base fuels used here contained very low levels of dissolved water, as shown in Table 2; B100 contained about 200 ppm, while the hydrocarbon fuels contained about 20 ppm. These fuels were exposed to excess water (a water layer underneath the fuel in a glass container), and the saturation water content was measured at 20 °C. For B100, this was over 1500 ppm, while results for the hydrocarbon fuels were around 60–85 ppm (see Table 2 and Table S-2 of the Supporting Information). Fuel producers and distributors generally make every effort to keep B100 nearly dry to avoid free water formation upon blending into hydrocarbon fuels.

### 3.2 Basic Fuel Properties of Biodiesel Blends

**Figure 1:**
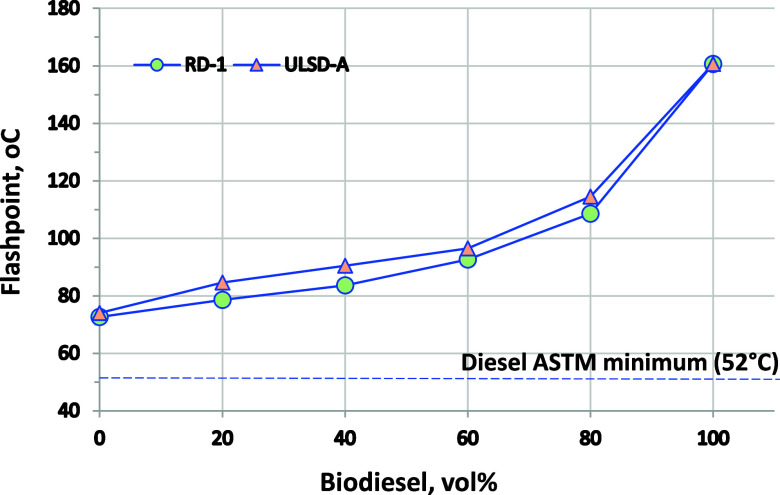
Flash point results for biodiesel blended into RD-1 and ULSD-A. ASTM D6450 repeatability is ±1.9 °C, which is smaller than the data points on this chart.

Flash point results are listed in Figure 1. A minimum flash point requirement ensures that the vapor above a fuel in storage (or onboard a vehicle) is not ignitable. The D975 specification for diesel requires a minimum of 52 °C, while the D6751 specification for B100 requires a minimum of 93 °C. Alternatively, producers can demonstrate a minimum of 130 °C flashpoint to avoid having to make a separate measurement to show that residual methanol is fully removed. RD-1 and ULSD-A have nearly the same flash point. The B100 flash point is very high at 160 °C, and the flash point increases as biodiesel is blended into both hydrocarbon fuels, generating nearly identical curves. The increase in flash point is not linear however, with an increasing slope above 60 vol %. There is no evidence of non-ideal solution behavior that could cause a flashpoint lower than that of the two blending components.^34^

**Figure 2:**
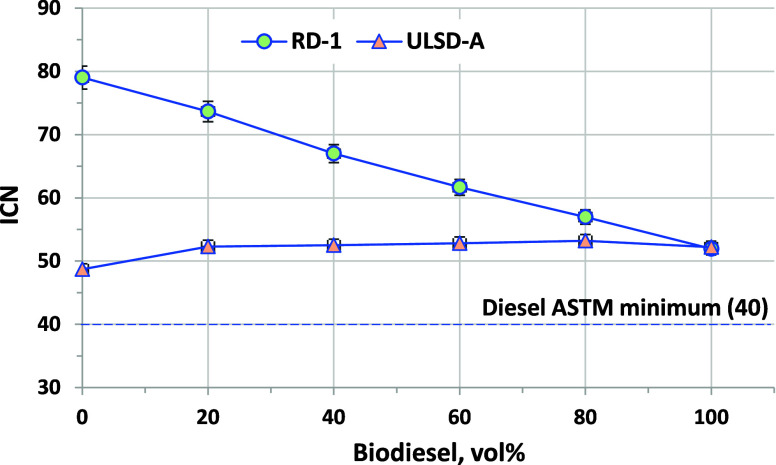
ICN results for biodiesel blended into RD-1 and ULSD-A. Error bars are ASTM D8183 repeatability and are, in some cases, smaller than the data points on this chart. Repeatability is approximately ±1 at 50 CN and ±1.8 at 80 CN.

CN is a measure of the reactivity of a fuel for autoignition, and a minimum CN ensures that diesel fuels will rapidly ignite upon injection into the engine. Because of complex kinetic interactions, many fuels do not blend linearly for CN or octane number, which is inversely related to CN.^35−37^ CN is shown in Figure 2 as an ICN measured by ASTM D8183, an approved alternative method that is highly correlated with conventional CN. ASTM D975 requires a minimum CN of 40, while D6751 requires a minimum of 45. The CN of RD-1 is 80, much higher than that of the B100 at 52. Upon blending with biodiesel, the CN decreases linearly. The CN of ULSD-A and B100 are only 3 CN units apart, and very little change in CN is observed at any blend level for these fuels.

**Figure 3:**
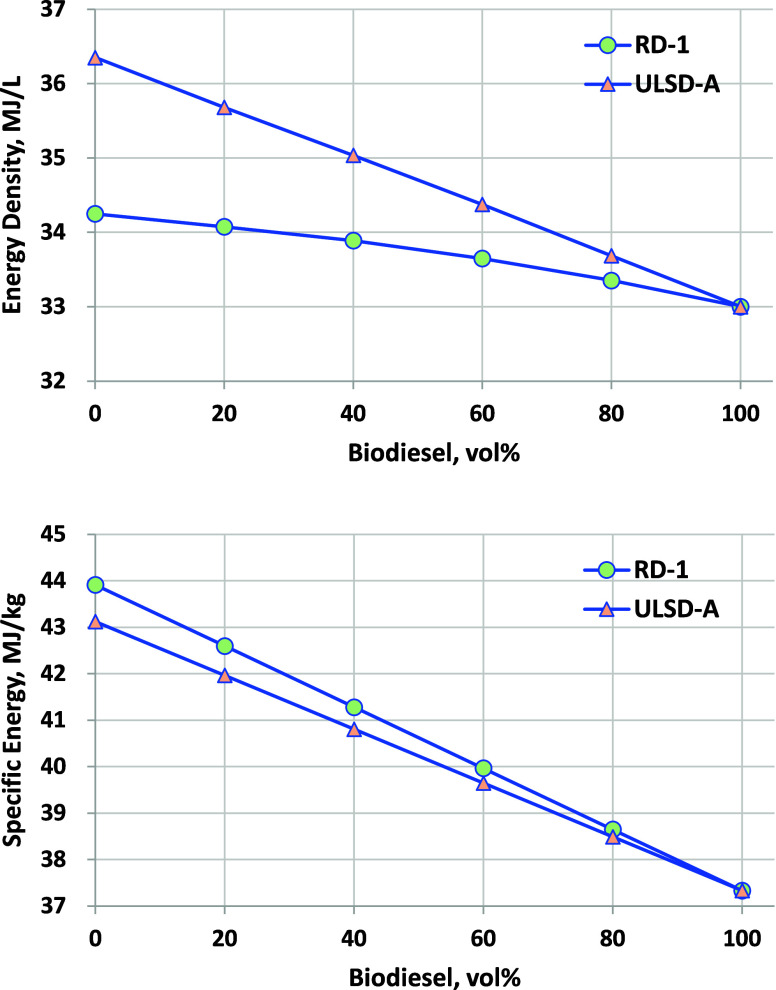
Lower heating value calculated from the blend component measured heating value and blend composition. (Top) Energy density (MJ/L); ASTM D240 D5291 combined repeatability for calculation of MJ/L from MJ/kg and density is ±0.18 MJ/L. (Bottom) Specific energy (MJ/kg); ASTM D240 and D5291 combined repeatability for calculation of the lower heating value from the higher heating value and weight percent H is ±0.22 MJ/kg.

Lower heating values can be discussed in terms of energy density (MJ/L) or specific energy (MJ/kg), and both are shown in Figure 3. Energy density of RD-1 is significantly lower (by 5.8%) than that of ULSD-A, with biodiesel being somewhat lower still (by 9.2% relative to ULSD-A). Blending of biodiesel results in a linear decrease in energy density. For specific energy, the value for RD-1 is 1.8% higher than that of ULSD-A. Biodiesel blending again results in a linear decrease in both hydrocarbon fuels, with B100 being 9% lower than ULSD and 4% lower than RD.

### 3.3 Low-Temperature Operability

**Figure 4:**
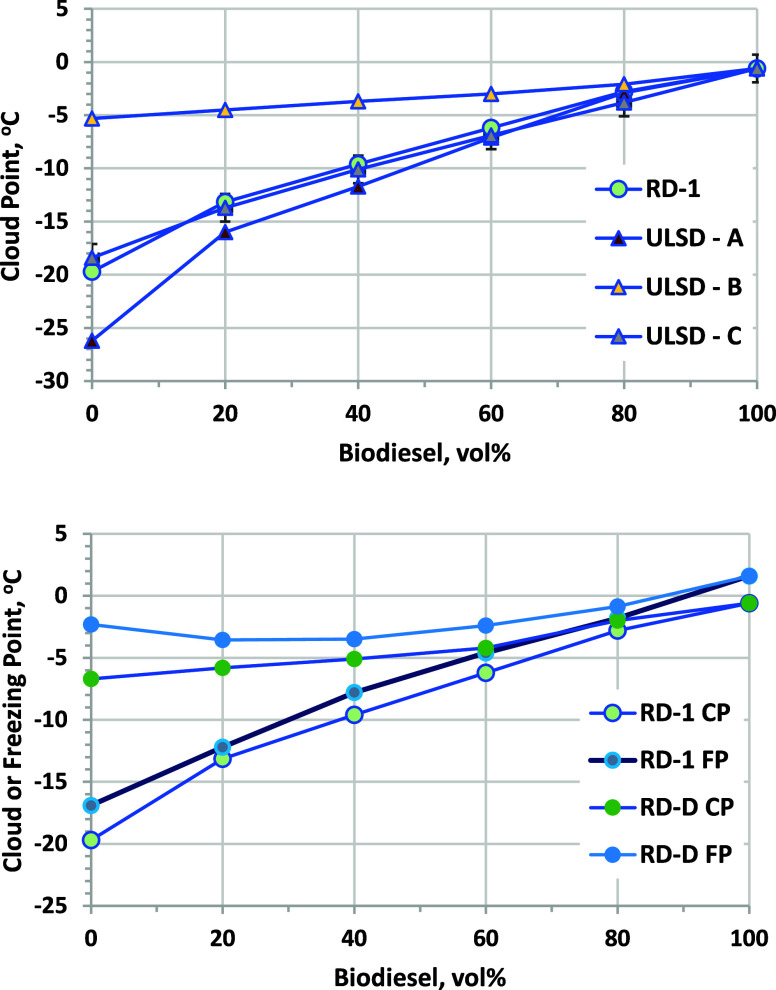
(Top) CP results for biodiesel blended into RD-1 and three ULSDs. Error bars are ASTM D5773 repeatability (±1.3 °C) and are shown only for the RD blends. (Bottom) Comparison of CP and FP results for biodiesel blended into RD-1 and RD-D samples. ASTM D5972 repeatability is ±0.5 °C and is not shown on the chart. Properties of RD-D are shown in Table S-1 of the Supporting Information.

Diesel fuels are reformulated to have a lower cloud point in areas that experience cold wintertime temperatures, and all fuel producers can be challenged to meet low-temperature operability requirements. A commonly used measure of low-temperature operability is the cloud point (CP), and results for RD and ULSD blends are shown in Figure 4. CP is measured by cooling the sample until the initial formation of crystals is observed. Because this crystallized material could clog an engine or fuel dispenser fuel filter is why CP is considered as the low-temperature operability limit. Finished fuel specifications (D975 and D7467) require that CP be reported but do not have specific limits because CP can be specified by the fuel distributor to meet local requirements. Results are shown for ULSD-A, which has a significantly lower CP than RD-1, and for ULSD-C, which has nearly the same CP as RD-1. Blending of only 20 vol % biodiesel in RD-1 and ULSD-B increases the CP by 5 °C, with CP increasing to −3 °C at 80 vol %. CP values for blends into RD-1 and ULSD-C are within the precision of the measurement, indicating no negative effects of the much lower polarity of RD versus ULSD on the solubility of saturated FAME components or other species that might precipitate at a low temperature.^38^ CPs are significantly lower for blends into ULSD-A, which has a much lower CP than ULSD-C. However, the benefits of blending into the lower CP ULSD-A vanish for blends of 60 vol % biodiesel and are higher relative to blending in ULSD-C.

A more conservative metric is the freezing point (FP), which is measured by cooling until crystals appear and then heating until crystals disappear, which normally occurs at a somewhat higher temperature than CP. Some marketers of biodiesel–RD blends use FP as their low-temperature operability metric for these fuels. FP values are roughly 2–3 °C higher than CP for these samples (Figure 4), providing a significantly more conservative estimate of the low-temperature operability limit. However, this does not consider the much slower cooling that occurs in real-world vehicle tanks and the long soaking times at a cold temperature, which can lead to crystal formation above CP in some cases.^38^ Given the potential for saturated FAME or biodiesel impurities, such as saturated monoglycerides, to precipitate over a period of hours at low temperatures, additional research is needed to fully understand RD–biodiesel blend low-temperature operability limits.

### 3.4 Surface Tension

**Figure 5:**
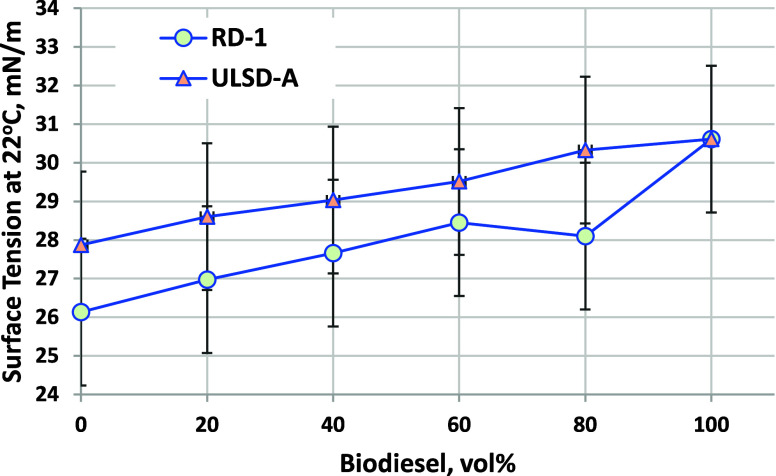
Surface tension at 22 °C for biodiesel blended into RD and ULSD-A. Error bars are D1331 method C repeatability of ±1.9 mN/m.

Surface tension results are listed in Figure 5. Surface tension is not a diesel or biodiesel specification property but impacts spray breakup and droplet formation (higher surface tension leads to larger droplets). Differences between RD, ULSD, and biodiesel blends mostly fall within the method repeatability; the differences are not significant. Nevertheless, discussing the trends that are observed, the surface tension of RD-1 at 22 °C is 26 mN/m, very similar to reported values for conventional diesel and jet fuels, which ranged from 26 to 29 mN/m.^19,39,40^ The surface tension of B100 was 30.6 mN/m, also similar to previously reported values.^40^ Upon biodiesel blending, the surface tension increases with an overall linear trend.

### 3.5 Density

**Figure 6:**
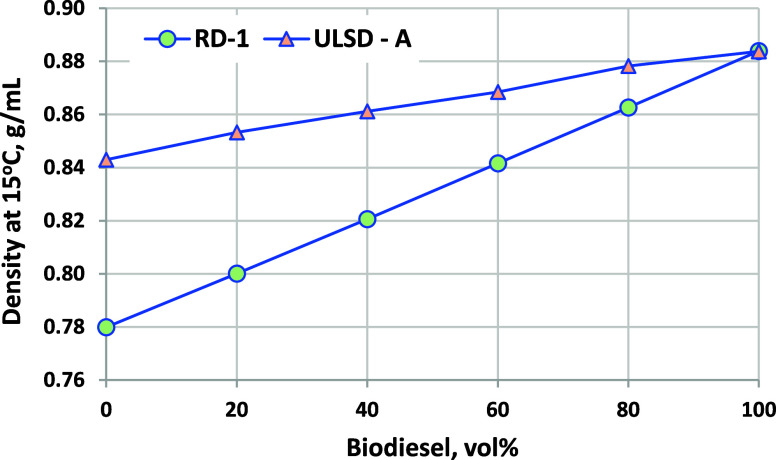
Density at 15 °C for biodiesel blended into RD-1 and ULSD-A. ASTM D7042 repeatability is ±0.0002 g/mL.

**Figure 7:**
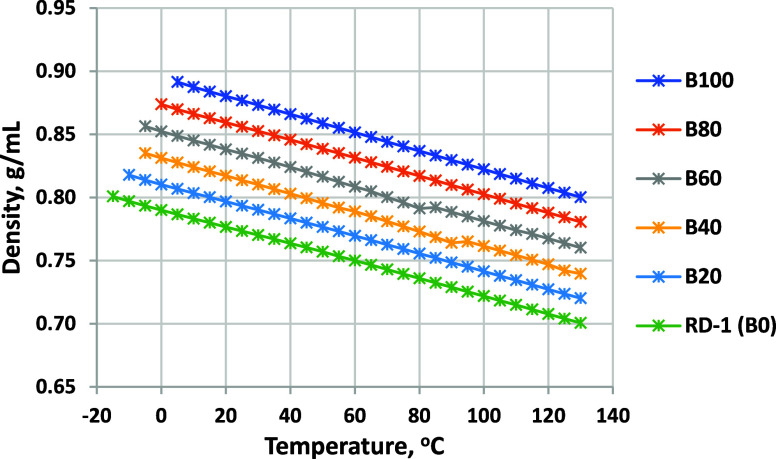
Density as a function of the temperature for biodiesel blended into RD-1 (minimum temperature for each fuel is limited by CP). ASTM D7042 repeatability is ±0.0002 g/mL.

**Figure 8:**
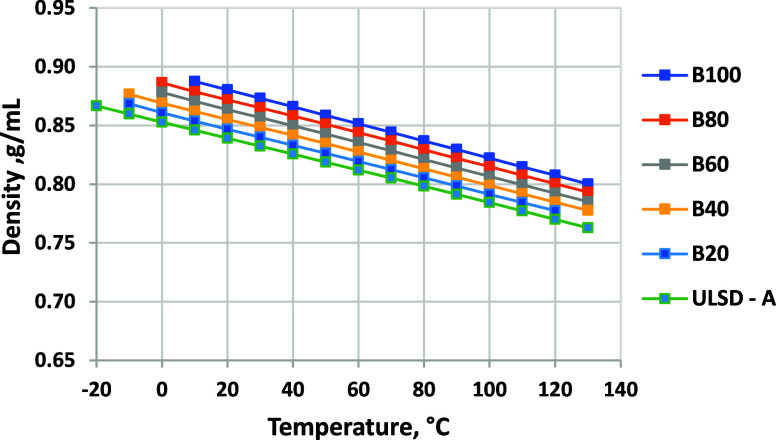
Density as a function of the temperature for biodiesel blended into ULSD-A (minimum temperature for each fuel limited by CP). ASTM D7042 repeatability is ±0.0002 g/mL.

Density is not a diesel or biodiesel specification property in ASTM standards but is used during product transfers to compensate for the temperature (fuels are sold on a volume basis, but flows are metered on a mass basis). Density can also impact spray atomization and penetration. Density blends highly linearly, as shown in Figure 6, with increasing density as biodiesel blend levels increase. Because of the lower density of RD-1 (and RD in general), the biodiesel–RD blend density is lower than that of neat ULSD-A up to 60 vol %. The high blend density values are well within the normal range for diesel fuels. Figure 7 shows density as a function of the temperature for the neat components and blends, which is also highly linear with the temperature and increases at a constant increment with every 20% biodiesel blended. Results for blends into ULSD-A are listed in Figure 8. Because of the higher density of the petroleum diesel, the density range between B0 and B100 at a given temperature is much smaller.

### 3.6 Kinematic Viscosity

**Figure 9:**
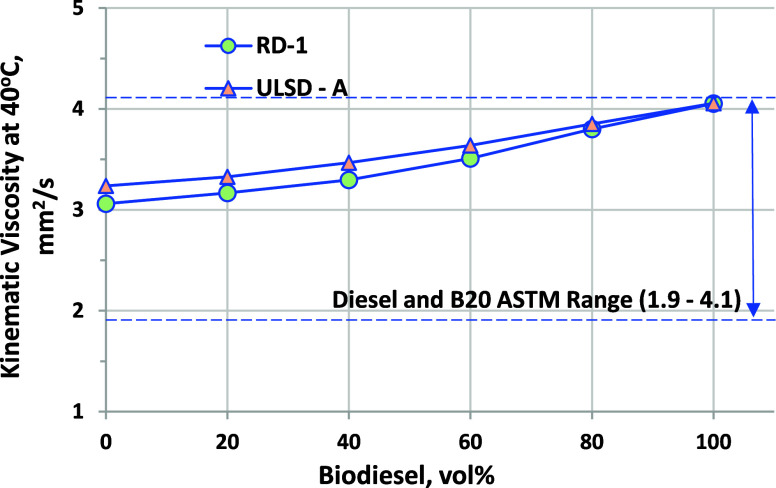
Viscosity at 40 °C for biodiesel blended into RD-1 and ULSD-A. ASTM D7042 repeatability is ±0.004 mm^2^/s.

**Figure 10:**
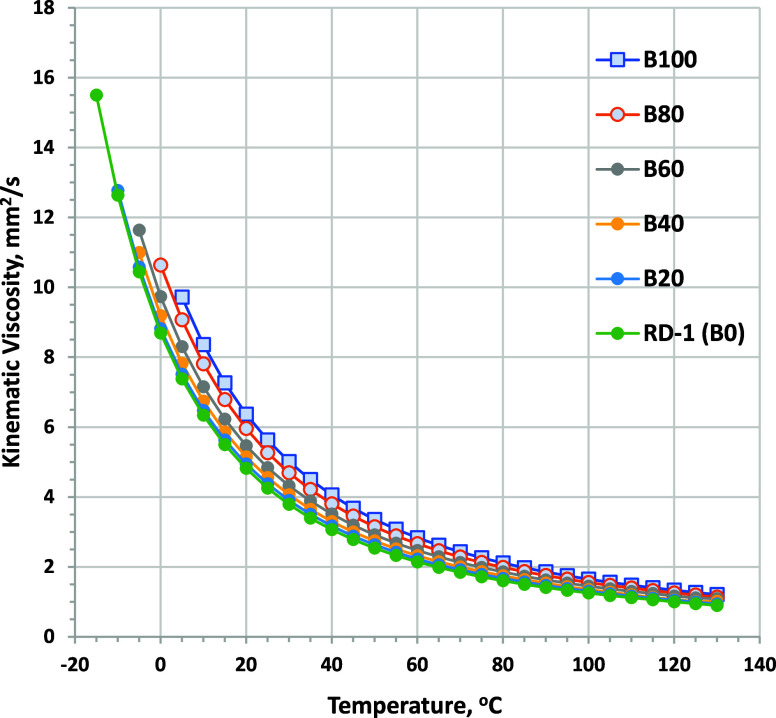
Kinematic viscosity as a function of the temperature for biodiesel blended into RD-1 (minimum temperature for each fuel is limited by CP). ASTM D7042 repeatability is ±0.004 mm^2^/s.

**Figure 11:**
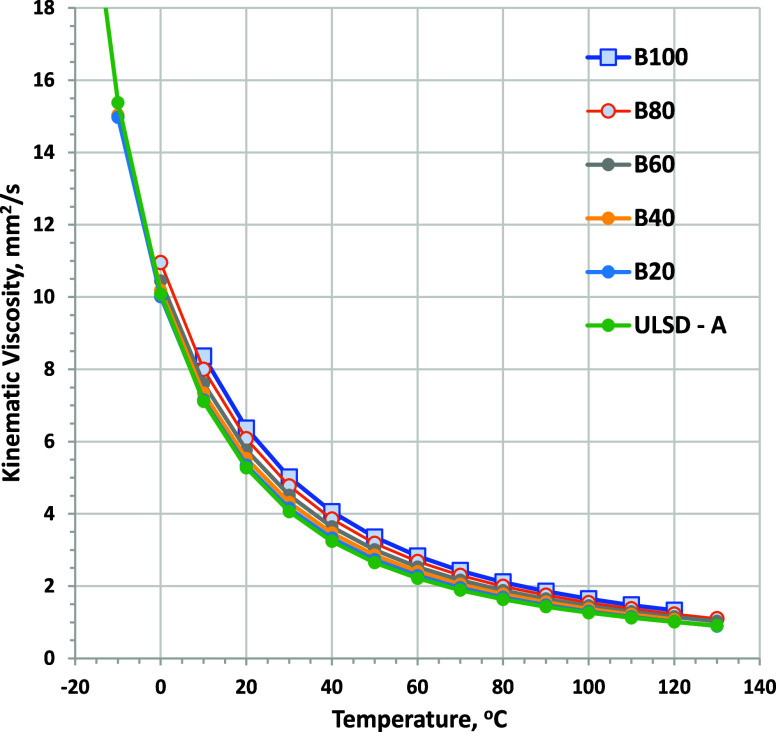
Kinematic viscosity as a function of the temperature for biodiesel blended into ULSD-A (minimum temperature for each fuel is limited by CP). ASTM D7042 repeatability is ±0.004 mm^2^/s.

Diesel engine fuel injection systems are designed to work with fuels falling in a specific range of viscosity, ensuring proper spray atomization and droplet formation as well as viscous lubrication of fuel pumps and injectors. Both D975 and D7467 limit kinematic viscosity to a minimum of 1.9 mm^2^/s and a maximum of 4.1 mm^2^/s at 40 °C. The limits for B100 in D6751 are 1.9–6.0 mm^2^/s, implying that, for biodiesel with kinematic viscosity over 4.1 mm^2^/s, viscosity could limit blending. Both RD-1 and ULSD-A blends are within the required limits and blend approximately linearly at 40 °C, as shown in Figure 9. Viscosity as a function of temperature curves for RD-1 and ULSD-A blends is shown in Figures 10 and 11, respectively. Viscosity becomes an increasingly strong function of the temperature as the temperature is reduced. All fuels evaluated show very similar trends with the temperature, although the viscosity range for blends with ULSD-A is narrower. At 0 °C, B80 blends could have viscosity as much as 2 mm^2^/s higher than that of RD-1. Biodiesel blending into ULSD-A causes a slightly lower viscosity increase. Note that the cloud point of the fuels limits the minimum temperature where viscosity can be measured.

### 3.7 Distillation

For diesel fuels (including RD), the only distillation parameter specified in ASTM standards is T90 or the temperature at which 90 vol % of the fuel has evaporated. Limiting T90 to a maximum of 338 °C (for No. 2 diesel fuel and RD) ensures that all of the fuel will evaporate and burn in the engine, because failure to evaporate can lead to in-cylinder carbon deposits and excessive lube oil dilution by the fuel. Biodiesel B6–B20 blends are allowed a 5 °C higher T90 limit of 343 °C on atmospheric distillation, because no carbon deposits or excess lube oil dilution have been observed for these fuels. Distillation requirements for blends above 20 vol % biodiesel have not been set. The distillation curve is important for other reasons as well. Conventional petroleum diesel typically has a wide boiling range, with a T90 – T10 difference (T90 – T10) of 100 °C or more. This provides low-boiling-temperature components that ensure ease of cold starting and diesel oxidation catalyst (DOC) light-off for particle filter regeneration.^41^

For diesel fuels and RD, distillation is performed at atmospheric pressure using the D86 method. T90 of B100 is inherently much higher than that for ULSD because of the size and structure of the methyl ester molecules. Typically, atmospheric distillation of B100 fails because the fuel decomposes before reaching T90 or T95. Therefore, a vacuum distillation method is used to determine T90 for biodiesel (D1160), which is limited to a maximum of 360 °C for B100. Vacuum distillation temperatures are corrected to atmospheric pressure for reporting results in this method. There is so little data available on high-level biodiesel blends that it is an open question as to whether vacuum distillation might be required for blends above some blend level.

**Figure 12:**
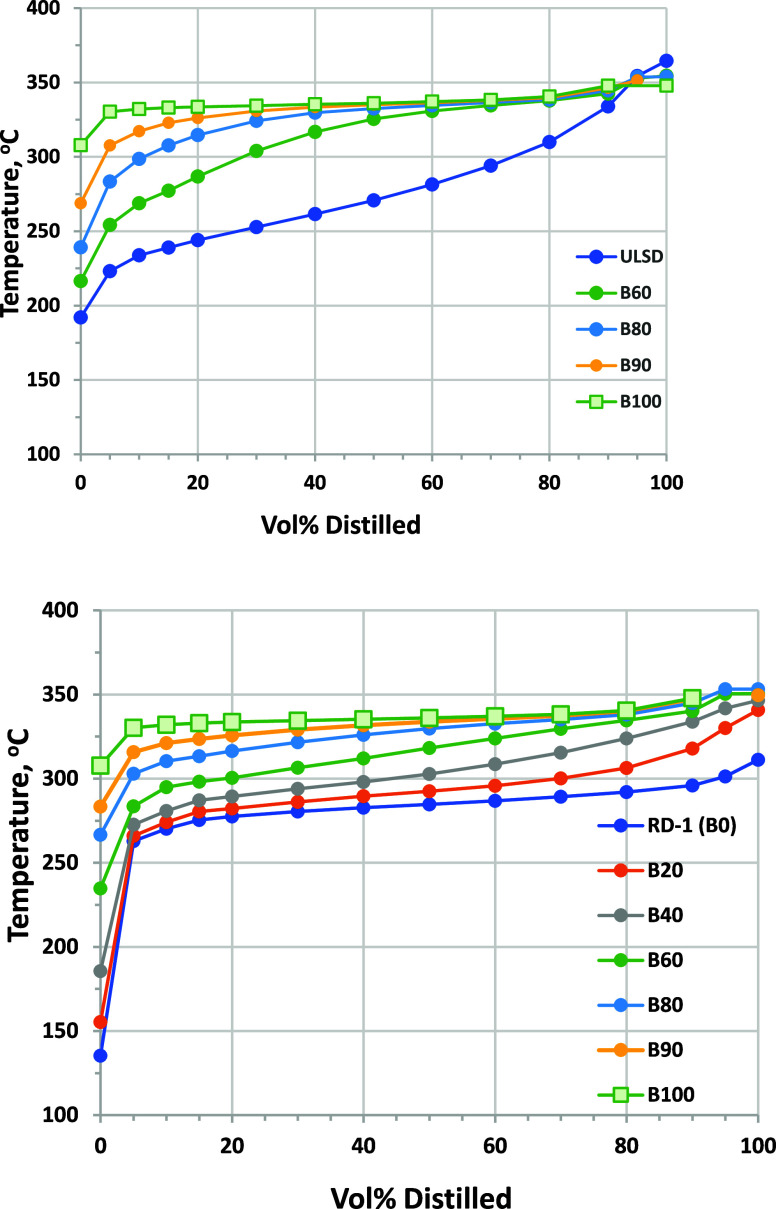
D86 distillation curves for ULSD and its biodiesel blends (top) and RD-1 and its biodiesel blends (bottom). ASTM repeatability for T10, T50, and T90 is ±1–2 °C, and ASTM repeatability for T95 is ±2–3 °C.

Distillation curves for B100 and blends into RD-1 and ULSD-A are shown in Figure 12. We used D86 atmospheric distillation for B100, and in this case, a T90 could be measured; however, the distillation ended before achieving T95. This experiment yielded a T90 value of 348 ± 2 °C, while vacuum distillation (D1160) yielded 352 ± 2 °C. It is notable that, for the B100, the difference between T90 and T10 is only 15 °C. The petroleum diesel, ULSD-A, has a relatively high T90 value of 334 °C compared to the specification limit of 338 °C, providing a near worst case scenario for this property in terms of blending biodiesel. ULSD-A also has T90 – T10 of 100 °C. RD-1 has a T90 of 296 °C, well below the maximum limit. RD-1 also has a relatively narrow distillation range, with T90 – T10 of only 26 °C, although this may not be true for all RD samples because Smagala and co-workers reported T90 – T10 values of 30–45 °C for RD from six different producers.^24^ We also measured values of 71 °C for RD-D and 37 °C for RD-G (Table S-2 and Figure S-1 of the Supporting Information).

Blending biodiesel into either hydrocarbon fuel increases the temperature of the distillation curve (Figure 12). For RD–B90, the distillation ended before achieving T95; however, the distillation did complete for ULSD–B90. Thus, we can conclude that, for blends up to at least B80, atmospheric distillation by the D86 method could successfully be used for the fuels examined in this study (considering RD–B90 distillation to have been unsuccessful). Blending of biodiesel into either hydrocarbon raises the T90, such that, at B60, it is almost equal to the B100 T90. However, T10 is not impacted as much, such that T90 – T10 increases up to B40 for RD or B60 for ULSD before declining (Figures S-2 and S-3 of the Supporting Information).

**Figure 13:**
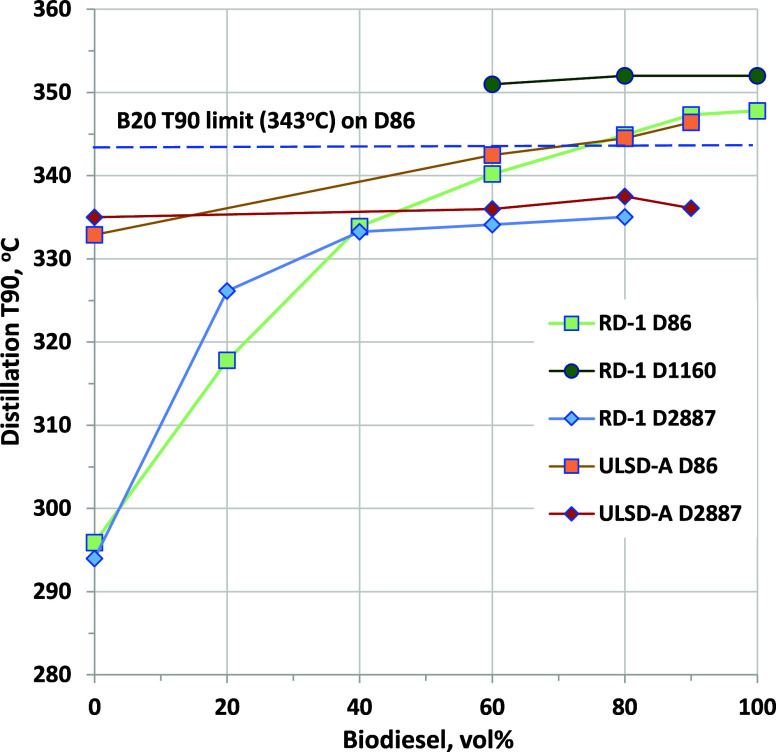
T90 from atmospheric distillation (D86), vacuum distillation (D1160), and GC-simulated distillation (D2887 – D86 correlation) for biodiesel blended into RD-1 and ULSD-A. ASTM D86 repeatability is roughly ±1.4 °C; ASTM D1160 is roughly ±2 °C; and ASTM D2887 is roughly ±0.7 °C, for this temperature range.

Distillation T90 results for RD, B100, and their blends using different methods are shown in Figure 13 (complete distillation curves are tabulated in the Supporting Information). As noted, the B100 and RD–B90 sample atmospheric distillations could not be completed because no additional liquid came off above T90, but T90 was measured as 348 and 347 °C, respectively, above the T90 limit for B20 of 343 °C. All other RD–biodiesel and ULSD–biodiesel blend samples were successfully distilled. As shown in Figure 12, B80 blends into either hydrocarbon fuel exhibited a T90 of 345 °C, slightly above the 343 °C limit for B20. If 343 °C can be considered a performance-based limit applicable to any fuel, then T90 may limit biodiesel blending to roughly 80 vol %.

Because atmospheric pressure distillation could not be completed for B100 or RD–B90, vacuum distillation may be required to fully understand the distillation properties of blends at these high levels. Vacuum distillation results for T90 are in Figure 13 for RD–B60 and higher blends (complete distillation curves are reported in the Supporting Information). All samples were distilled at a 50 mmHg pressure. For B100, D1160 yielded a value of 352 °C, with essentially the same result obtained for the B60 and B80 blends, which were both adequately distilled using D86. While the T90 for B100 from D86 and D1160 may be within measurement error, values of T90 from D86 are much lower for B60 and B80 blends. It seems likely that D1160 should be used only for samples that cannot be distilled at atmospheric pressure.

For diesel, RD, and biodiesel blends up to 20 vol %, simulated distillation by GC (D2887 – D86 correlation) provides results that have been shown to be equivalent to D86 distillation. Results of simulated distillation using a D2887 – D86 correlation are also reported in Figure 13. This method is only validated to provide accurate results for biodiesel blends into conventional diesel up to 20 vol %. Results for RD-1 and ULSD-A are very close to the physical distillation values. We find an 8 °C difference between D86 and D2887 for RD-1 B20, and results for B60 or higher blends significantly underestimate the physical distillation value. For ULSD-A blends at 60, 80, and 90 vol % biodiesel, we also observed values that are much lower than those from physical distillation. While there may be potential for the T90 of these higher level blends to be accurately quantified, additional development of the GC method and the D86 correlation will be required.

### 3.8 Oxidation Stability

While petroleum diesel and RD are generally regarded as being stable in storage, oxidation (or storage) stability is a critical property for maintaining biodiesel and biodiesel blend quality during transport and distribution. Oxidation reactions occur between susceptible functional groups in the fuel and dissolved oxygen. In biodiesel, the susceptible functional groups are bis-allylic C–H bonds in polyunsaturated fatty acid chains.^42,43^ Peroxides form initially, and as their concentration increases, they can decompose to form organic acids and aldehydes or dimerize. The aldehydes can also be polymerized to form insoluble gums. These degradation products lead to increased corrosivity, increased viscosity, as well as deposits in fuel pumps and injectors.^43^ The most common approach to managing biodiesel and blend stability is treatment with antioxidant additives, such as butylated hydroxytoluene (BHT) or *tert*-butylhydroquinone (TBHQ).^44^

Because of the low temperature and low oxygen concentration, oxidation in the field takes place over a period of weeks to months.^45−47^ Therefore, highly accelerated tests are used to provide an estimate of the oxidation stability in a matter of hours. The test used for B100 and B6–B20 blends is Rancimat (also known as the oil stability index). In this test, air is bubbled through the fuel at a high space velocity at 110 °C. Under these high-temperature and oxygen-replete conditions, antioxidant is consumed, and then the fuel rapidly oxidizes, producing aldehydes that decompose to yield volatile organic acids (mainly formic, acetic, and caproic acids).^48^ These are swept out of the fuel by air, which then passes through a water bath whose conductivity is monitored. Upon dissolution of the acids in water, conductivity rapidly increases. The time from the start of the experiment to this sharp increase in conductivity is the Rancimat induction time, which is required to be over 3 h for B100 and over 6 h for B6–B20 blends. The 3 h value for B100 is intended to ensure that B20 blends will be over 6 h. The Rancimat test is quite specific to the oxidation of single, bis-allylic, and conjugated double bonds, as found in fats and oils as well as biodiesel made from them. Materials that oxidize by other mechanisms, such as diesel boiling range ethers,^35,49^ petroleum-derived jet fuels,^50^ or RD/ULSD, cannot be assessed for stability using this method in our opinion.

**Figure 14:**
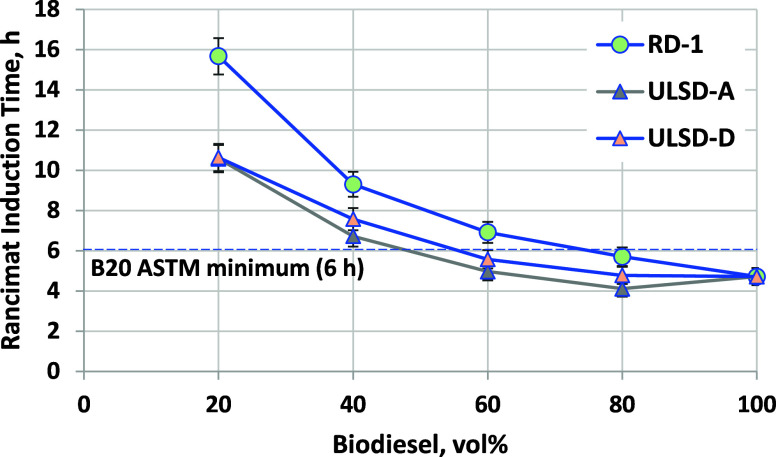
Rancimat induction time results for biodiesel blended into RD-1, ULSD-A, and ULSD-D. Error bars are method EN 15751 repeatability.

Figure 14 shows Rancimat induction time results for B100 and biodiesel blends with RD-1, ULSD-A, and ULSD-D (see Table S-3 of the Supporting Information for the ULSD-D properties). The B100 induction time of 4.7 h is well above the minimum requirement in D6751 of 3 h. The B20 blends in RD-1 had an induction time of 16 h, above the minimum 6 h required in D7467. While there are no specifications for higher level blends (above 20 vol %), this 6 h requirement could possibly be considered as a performance requirement, meaning that it could be applied to any blend level of biodiesel. The B40 and B60 blends meet this requirement, but the B80 blend fails. This indicates that, for blending B80, B100 with a higher induction time is required, likely achieved by blending of higher antioxidant levels. B100 in the U.S. market has had an average 9 h Rancimat induction time in recent years,^51^ which would likely be adequate for achieving greater than 6 h in the RD-1 B80 blend. The results shown here for a 4.7 h induction time of B100 are close to a worst case scenario.

Also shown in Figure 14 are Rancimat induction time results for biodiesel blends into ULSD-A and ULSD-D, which were chosen because of their marginally low stability in the PetroOxy test (as discussed below). These blends are significantly less stable than the RD-1 blends but still over 6 h for B20 and B40. Higher blends did not meet the 6 h minimum, although with B100 at the current market average of 9 h, it seems likely that these blends would pass. Note that the difference in stability for blends into different base hydrocarbon fuels diminishes with increasing biodiesel blend levels.

**Figure 15:**
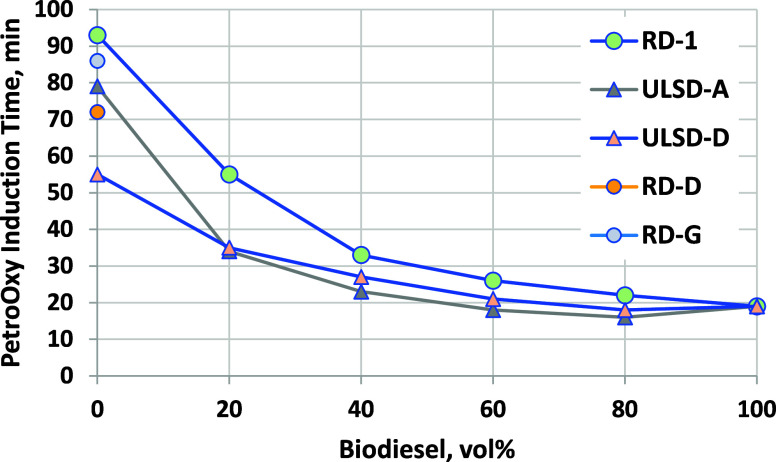
PetroOxy induction time results for biodiesel blended into RD, ULSD-A, and ULSD-C. ASTM D7545 repeatability ranges from ±1 to 3 min for induction times in this range. Results for additional RD samples (RD-D and RD-G, with properties shown in Table S1 of the Supporting Information, 0% biodiesel) are shown for context.

Oxidation stability was also measured using ASTM D7545, known as the PetroOxy method or the rapid small-scale oxidation test. In this method, the sample is placed in a pressure vessel that is pressurized to 700 kPa with oxygen at ambient temperature and then heated to 140 °C. The pressure is monitored over time until a 10% drop from the maximum pressure is observed, which is defined as the breakpoint or PetroOxy induction time. Because this test is based on oxygen consumption, it may be more generally applicable than Rancimat; that is, the stability of materials that do not contain bis-allylic or conjugated double bonds may also be assessed. This test is also much faster than Rancimat, making it attractive to fuel producers and distributors. Many engine manufacturers recommend the use of Top Tier diesel fuel, which requires a minimum of 60 min PetroOxy induction time for B0–B2 blends (for higher blends up to B20, Top Tier recommends a 20 h minimum Rancimat induction time, which would require a higher stability B100 than examined here).^52^ PetroOxy induction times for the RD, B100, and blends evaluated in this study are shown in Figure 15. RD-1 is highly stable with an induction time of 93 min. Induction times decrease with biodiesel blending, with B20 being just below the 60 min Top Tier diesel minimum. Blends into ULSD-A and ULSD-D were less stable and showed a similar decreasing trend. Induction times for two additional RD samples (B0) are shown for context (for properties of these, see Table S-2 of the Supporting Information).

**Figure 16:**
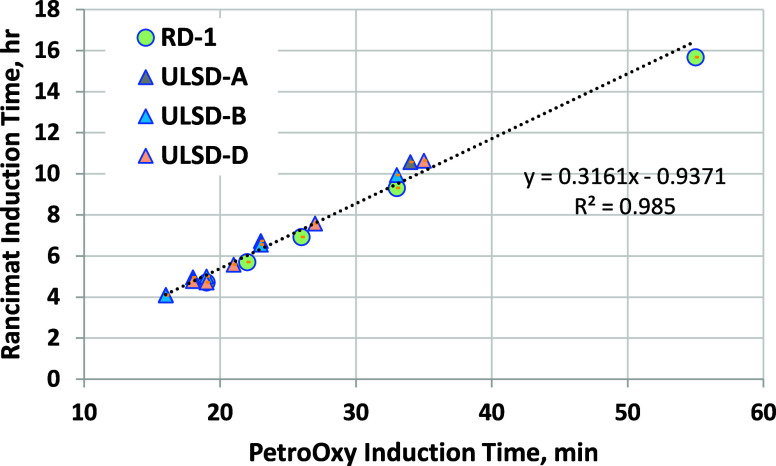
Correlation between the Rancimat induction time and PetroOxy induction time for biodiesel blends.

The correlation between PetroOxy and Rancimat induction times is very strong for this limited data set (Figure 16), something that has been observed in previous studies^53,54^ with small sets of related samples. However, the methods are not strongly correlated when data from multiple studies and diesel/biodiesel samples are included.^55^ This is believed to be caused by biodiesel containing different antioxidants oxidizing at different rates in the two tests. Nevertheless, for the samples evaluated here, the Top Tier PetroOxy requirement of 60 min appears to be a much higher level of stability than that required by ASTM standards. The D7467 6 h minimum for B6–B20 blends corresponds to a PetroOxy induction time of only 22 min, while the 60 min PetroOxy induction time corresponds to a Rancimat induction time of roughly 18 h.

## 4 Discussion and Conclusion

This study examined the impact of biodiesel blending on properties of conventional diesel (ULSD) and RD. The focus was on understanding if there were differences in the impact of biodiesel blending for the two hydrocarbon blendstocks, in understanding what properties might limit the biodiesel blend level that can be achieved while retaining the expected performance, and identifying research needs for achieving high blends or operation on 100% low-carbon fuels. In general, property impacts of biodiesel blending were not different for blending into RD in comparison to ULSD, for the fuels and properties examined here. Thus, the potential blending limitations of certain properties apply to both hydrocarbon fuels.

Flashpoint, CN, LHV, surface tension, and density do not appear to have any potential to limit biodiesel blending. Biodiesel generally has a higher flashpoint than RD or ULSD; therefore, blending increases flashpoint. It also has a higher CN than ULSD but lower CN than RD. The lower heating value on an energy density basis (MJ/L) was 9% lower for biodiesel relative to ULSD and 4% lower relative to RD, such that the energy content of the blended fuels is marginally lower. Surface tensions of biodiesel, RD, and ULSD all fell within the range of values observed for ULSD. Density increased linearly with biodiesel blending into both RD and ULSD.

Low-temperature operability (cloud point), viscosity, distillation curve, and oxidation stability all have the potential to limit biodiesel blending, at least in some situations.

CP is an inherent physical property of fuels that cannot be changed by the use of fuel additives. All distillate fuel producers reformulate fuel products for use in cold wintertime temperatures. Flow improver additives can prevent fuel filter clogging and extend operability to a few degrees below the CP. Biodiesel tends to have a significantly higher CP than wintertime ULSD. For ULSD and RD with the same CP, we observed no difference in the effect of biodiesel blending. Thus, there was no impact of the lower polarity of RD on the solubility of FAME or impurities, such as monoglycerides. Because of occasional field reports that filter clogging issues may occur above CP for RD biodiesel blends, we examined the FP as a more conservative metric. FP values are roughly 2–3 °C higher than CP for these samples (Figure 4), providing a significantly more conservative estimate of the low-temperature operability limit.

**Figure 17:**
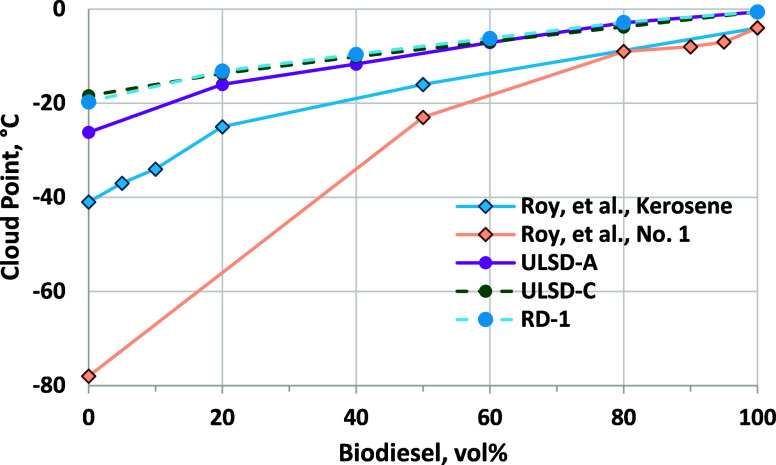
CP results from this study and for blending into a No. 1 diesel and kerosene (data from ref (56)). Repeatability for ULSD and RD blends is ±1.3 °C. CP from Roy et al. employed ASTM D7689 with repeatability of ±0.6–1.4 °C in the 0 to −40 °C range.

Biodiesel blend CP can be managed by reducing blend levels or blending into lower CP hydrocarbon blendstocks, such as No. 1 diesel or kerosene, during the winter months. No. 1 diesel is a lighter distillation cut than No. 2 diesel, leading to a significantly lower CP. There are many published studies of biodiesel blending into kerosene that are mainly focused on combustion and emission effects, but few report CP results. An exception is the study by Roy et al., who prepared blends of a canola biodiesel (−4 °C CP) with a Canadian winter diesel and a kerosene.^56^ The winter diesel had a CP of −41 °C and other properties that indicate that it can be considered a No. 1 diesel fuel. The kerosene had an even lower CP of −78 °C. CP results for biodiesel blends in these fuels, along with CP results from Figure 4, are shown in Figure 17. Clearly blending into No. 1 or a very low CP kerosene can improve low-temperature properties of intermediate blends, but as observed in Figure 4 and even for blending into −78 °C CP kerosene, at some blend level, the base fuel effect diminishes. Thus, to achieve blend levels as high as B60 or B80, this approach is not likely to be as effective.

Another approach that has been successfully used is retrofitting the engine with a heated fuel system that allows use of B100 year-round.^57^ On a longer term, biodiesel could be processed or biodiesel feedstocks could be modified to eliminate saturated fatty acids,^58^ contain branched fatty acids,^59^ or have shorter chain fatty acids with lower melting points.^60^

While not specifically examined in the experiments reported here, a second factor that can limit biodiesel blending is the kinematic viscosity. The upper limit for viscosity for diesel fuel and blends up to B20 is 4.1 mm^2^/s at 40 °C. The limits for B100 in D6751 are 1.9–6.0 mm^2^/s, implying that, for biodiesel with kinematic viscosity over 4.1 mm^2^/s, viscosity could limit blending. B100 used in this study had viscosity of 4.06 mm^2^/s; however, several studies that evaluated B100 samples from various feedstocks observed kinematic viscosity well above this level.^61,62^

Perhaps the most important factor that may limit biodiesel blending is the distillation curve, in terms of both T90 and the distillation range (T90–T10). D86 atmospheric distillation was successfully used to measure T90 for all blends up to 80 vol % but not consistently for B90. ULSD-A used in this study had a relatively high T90 of 334 °C compared to the D975 T90 limit of 338 °C. B20 blends are allowed for a higher T90 of 343 °C. We observed that B80 blends in either hydrocarbon fuel had a T90 of 345 °C, even though the T90 of the RD was only 296 °C. Clearly, the effect of the T90 of the base hydrocarbon fuel diminishes or becomes insignificant as the biodiesel blend level increases. These results suggest that 80 vol% biodiesel may be an approximate upper limit if the B20 limit of 343 °C can be considered a performance-based T90 limit applicable to any biodiesel blend.

There is also evidence that the higher temperature and narrower boiling point range (T90–T10) of high-level biodiesel blends can cause failure of DOC light-off. In these systems, supplemental fuel is injected into the exhaust to be oxidized over the DOC, increasing the exhaust temperature for particle filter regeneration. A recent study of high-level blends found that B50 and higher blends were not being converted over the DOC at temperatures below about 350 °C, likely because most of the fuel was not evaporating and simply passed through the catalyst as aerosol droplets.^41^ A system that employs a biodiesel blend level sensor may allow for calibration of the engine emission control system to take the properties of biodiesel into account. While there are many papers describing blend level sensors,^63,64^ little has been published on integration of a sensor with emission control system operation. Also, a fuel with a higher fraction of lower boiling components, such as No. 1 diesel or kerosene, might mitigate this issue.

**Figure 18:**
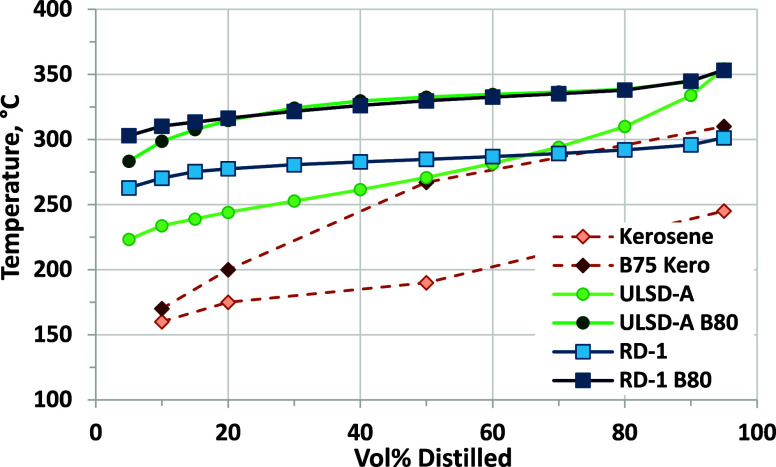
Comparison of distillation curves from this study with results for a B75 kerosene blend from ref (65). ASTM D86 repeatability for T10, T50, and T90 is ±1–2 °C, and ASTM D86 repeatability for T95 is ±2–3 °C.

As noted above, there are many studies focused on combustion and emission impacts of blending biodiesel with No. 1 diesel or kerosene, but few report detailed properties. However, Aydin has reported distillation data for safflower-oil-derived biodiesel into kerosene, and it is interesting to examine these results alongside results from the current study.^65^ This comparison is shown in Figure 18 for B80 blends from this study and B75 blends in kerosene. For ULSD-A, 80% biodiesel increases distillation temperatures across the distillation curve, essentially matching the B100 distillation at about B50, as shown in Figure 12. RD-1 has a much lower T90 than ULSD-A but much higher T10, such that the B80 distillation curve is almost the same as ULSD-A B80. The kerosene T10 value is 74 °C lower than that of ULSD-A and 110 °C lower than that of RD-1. Blending of 80% biodiesel into the kerosene increases distillation temperatures but retains a wide distillation range with T95 – T10 of 140 °C compared to values of 55 °C for ULSD-A and 43 °C for RD-1 B80 blends. T90 (or T95 as reported by Aydin) is also significantly lower than those for the ULSD and RD blends. These results support the idea that using No. 1 diesel or kerosene as the hydrocarbon blendstock can mitigate distillation limitations on biodiesel blending.

For the accelerated oxidation stability tests used here, there were no unexpected differences between blends of biodiesel into RD versus ULSD. Blends of RD-1 were slightly more stable than blends in ULSD-A, likely because RD-1 is slightly more stable than ULSD-A (note that this observation is specific to RD-1 and the ULSD samples examined here and should not be considered a general conclusion). The impact of hydrocarbon blendstock stability diminished as more biodiesel was blended. These results suggest that, to achieve the same level of stability as, for example, a B20 blend, higher level blends will require higher antioxidant treatment rates. Oxidation stability may limit blending if extremely high levels of antioxidant are required as blend levels increase; however, at least for these accelerated tests, that does not seem to be the case. While blends up to B40 met the 6 h minimum Rancimat required for B20, experience suggests that, with a more stable B100, more typical of today’s market fuels, blends up to B80 could also meet this requirement. This argument depends upon the idea that a 6 h Rancimat is a performance-based limit that applies to all blend levels, which needs to be verified experimentally or in field studies.

The fact that the blending of biodiesel can increase density, viscosity, surface tension, and boiling point (or T90) leads to the possibility that spray atomization and fuel mixing/evaporation will be degraded. To some extent, this is mitigated by the very high injection pressures utilized in modern diesel engines (potentially up to 300 MPa). Degraded spray atomization could lead to higher engine-out soot emissions, increased fuel wall wetting and lube oil dilution, and poor combustion efficiency. Research should be conducted to determine the extent to which this is a realistic concern. This may primarily be of concern during cold starting, in which case the use of a heated fuel system could also mitigate this issue.

On the basis of these findings and analysis, the following research is recommended:

- Given the potential for saturated FAME or biodiesel impurities, such as saturated monoglycerides, to precipitate over a period of hours at low temperatures, additional research is needed to fully understand RD–biodiesel blend low-temperature operability limits.
- Additional research is needed to understand how the high T90 and very low distillation range of B100 and high-level biodiesel blends impact lube oil dilution, engine deposits, and diesel oxidation catalyst light-off. Research should also determine if the T90 limit for B20 of 343 °C can be applied to higher biodiesel blend levels.
- Criteria need to be established for when atmospheric distillation versus vacuum distillation can be used to determine T90 for high-level blends.
- While simulated distillation accurately predicted T90 on atmospheric distillation for RD and ULSD samples, it was less successful for RD blends and for any blend of 40 vol % or higher. Because of the convenience of simulated distillation, research to determine if this method can be extended to high-level biodiesel blends is of great interest.
- The use of blend-level sensors and biodiesel-specific engine/emission control system calibrations deserves extensive investigation.
- A better understanding of how the use of No. 1 diesel or kerosene impacts CP and the distillation curve of high-level biodiesel blends needs to be developed. There may be benefits to a lower boiling hydrocarbon blendstock that is formulated specifically for blending with biodiesel at high blend levels.
- Additional research is needed to define stability levels in terms of Rancimat or potentially PetroOxy induction times required for higher biodiesel blends and to determine if the 6 h Rancimat requirement for B20 is adequate for higher level blends. This research should be based on long-term storage tests, such as ASTM D4625.

## Supporting Information Available

Acronyms and abbreviations, details on test methods, properties of three commercial renewable diesel and four commercial ULSD samples, and full distillation curves for fuels evaluated (PDF)
