# Supplementary material for: Properties That Potentially Limit High-Level Blends of Biomass-Based Diesel Fuel
Source: Energy Fuels. 2024 May 7;38(10):8829–41. doi: 10.1021/acs.energyfuels.4c00912 (PMC11103708; doi:10.1021/acs.energyfuels.4c00912)
Supplement: Supplementary file 1 — ef4c00912_si_001.pdf [file ef4c00912_si_001.pdf]

Supporting Information

Properties that potentially limit high level blends of biomass-based diesel fuel

Robert L. McCormick*, Gina M. Fioroni, Nimal Naser, Jon Luecke

National Renewable Energy Laboratory
15301 Denver West Parkway, Golden, CO 80401 United States

Contents

1 Acronyms and abbreviations
2 Details on test methods
3 Properties of three commercial renewable diesel and four commercial ULSD samples
4 Full distillation curves for fuels evaluated

List of Tables

Table S-1. Specific instruments models used to measure fuel properties

Table S-2. Properties of ultra-low sulfur diesel fuels used in this study.

Table S-3: Properties of commercial renewable diesels

Table S-4: Atmospheric (D86) distillation curves for RD samples

Table S-5: GC simulated (D2887 – D86 correlation) distillation curves for RD samples

Table S-6: Atmospheric (D86) distillation results for RD and biodiesel blends

Table S-7: Atmospheric (D86) distillation results for ULSD – C and biodiesel blends

Table S-8: Vacuum (D1160) distillation results for RD and biodiesel blends

Table S-9: GC simulated (D2887 – D86 correlation) distillation results for RD and biodiesel blends

Table S-10: GC Simulated (D2887 – D86 correlation) distillation results for ULSD – C and biodiesel blends

List of Figures

Figure S-1. T90-T10 and T50-T10 values for biodiesel blends into ULSD-A

Figure S-2. T90-T10 and T50-T10 values for biodiesel blends in to RD

Figure S-3. D86 Distillation curves for RD, RD-D, and RD-R.

* Corresponding author: robert.mccormick@nrel.gov, Phone: +01 303 275 4432

1 Acronyms and abbreviations

ASTM	ASTM International, a standards setting organization
Bxx	A biodiesel blend containing xx vol% biodiesel
CN	Cetane number
CP	Cloud point
DOC	Diesel oxidation catalyst
EN	European Normalization
GC	Gas chromatograph
ICN	Indicated cetane number (an approved alternative method for measuring CN)
JP-5	Military jet fuel used by the U.S. Navy
RD	Renewable diesel
Txx	Temperature for xx vol% of a sample to evaporate in a distillation experiment
ULSD	Ultra-low sulfur diesel

2 Details on test methods

Table S-1. Blend Property Measurement Methods

Property	Method	Sample Volume, mL	Equipment
Kinematic viscosity, mm^2/s	D7042	5	Anton Parr SVM3001
Density, g/mL	D7042	5	Anton Parr DMA4200M
Surface tension, mN/m	D1331	20	Biolin Scientific Sigma 701
Flash point, °C	D6450	2	Eralytics Eraflash
Cloud point/freeze point, °C	D5773/D5792	<1	Phase technology FCA-70X
Distillation T90, °C	D86	200	Petroleum Analyzer Company (PAC) Model: OptiDist
Distillation T90, °C	D1160		Herzog Model: HDV 632
Distillation T90, °C	D2887	<1	Agilent 7890A gas chromatograph, SimDis Expert software version 9.39 Advance
CN	D8183	40	ASG Advanced Fuel Ignition Delay Analyzer (AFIDA) 2805
Lower heating value, MJ/kg	D240	2	IKA C2000 Combustion Calorimeter
Water content and saturation water solubility, ppm	D6304	2	Metrohm 831 KF Coulometer
Lubricity, microns	D6079		PCS Instruments: HFRR
Oxidation stability, h	EN15751	10	Metrohm 873 Biodiesel Rancimat
Oxidation stability, min	D7545	5	Petrotest PetroOxy

3 Properties of three commercial renewable diesel and four commercial ULSD samples

Table S-2. Properties of commercial renewable diesels

Property	Method	Renewable Diesel - 1[a]	Renewable Diesel - D	Renewable Diesel - G
Flashpoint, °C	D6450	72	65	73
Cloud point, °C	D5773	-19.6	-6.7	-9.5
Distillation T90, °C	D86	296	295	302
Distillation T90, °C	D2887	294	294	296
T90 – T10, °C	D86	25.6	71.3	37.2
Kinematic viscosity 40°C, mm^2/s	D7042	3.062	2.798	3.226
Density 15°C, g/mL	D7042	0.780	0.782	0.785
Water content, ppm[b]	D6304	16/85	15/61	15/57
Cetane Number (as ICN)	D8183	79	75	83
Sulfur, ppm	D5453	<0.5	<0.5	<0.5
Hydrogen, wt%	D5291	14.82	14.62	14.61
Lower heating value, MJ/kg	D240	43.91	44.14	43.91
Oxidation stability, min	D7545	93	72	86
Lubricity, micron	D6079	410[c], 590[d], 350[d]	--	--

[a]Used for blending in this study. [b]As received/saturation [c]As received. [d]After treating with silica gel to remove lubricity additive. [e]After silica gel treating and blending with 5% biodiesel.

Table S-3. Properties of ultra-low sulfur diesel fuels used in this study.

Property	Method	ULSD-A	ULSD-B	ULSD-C[a]	ULSD-D
Flashpoint, °C	D6450	73		--	80.5
Cloud point, °C	D5773	-26.2	-5.3	-18.4	-15.1
Distillation T90, °C	D86	333.9	337.5	--	330[c]
T90-10, °C	D86	100.1		--	98.2[c]
Kinematic viscosity 40°C, mm^2/s	D7042	3.239	--	--	
Density 15°C, g/mL	D7042	0.843		--	0.863
Surface tension 20°C, mN/m	D1331	27.872	--	--	--
Water content, ppm[b]	D6304	26/80	--	--	--
Cetane Number (as ICN)	D8183	48.7	51.8	--	--
Sulfur, ppm	D5453	5.3	--	--	9.4
Hydrogen, wt%	D5291	13.34	13.5	--	12.79
Lower heating value, MJ/kg	D240	43.12	--	--	42.79
Oxidation stability, min	D7545	79	69	--	55
Aromatics, vol%	GCxGC	27.8	27.5	--	--

[a]85 vol% ULSD-A + 15 vol% ULSD-B, [b]As received/saturation, [c]D2887

4 Full distillation curves for fuels evaluated

Table S-4: Atmospheric (D86) distillation curves for RD samples, °C

	**Renewable Diesel - 1[a]**	**Renewable Diesel - D**	**Renewable Diesel - G**
IBP	135.3	146.4	165.7
5%	262.9	193.7	244.8
10%	270.3	223.2	265.0
15%	275.4	243.2	273.3
20%	277.5	255.7	278.3
30%	280.6	269.7	285.0
40%	282.8	277.3	288.4
50%	284.8	281.8	291.0
60%	286.9	285.2	293.2
70%	289.2	287.8	295.4
80%	292.0	290.7	298.0
90%	295.9	294.5	302.2
95%	301.3	298.3	311.9
FBP	311.3	308.5	326.6
Recovered, mL	97.1	98.0	97.3
Residue, mL	1.6	1.3	2.3
Loss, mL	1.3	0.7	0.4

[a]Used for blending in this study.

Table S-5: GC simulated (D2887 – D86 correlation) distillation curves for RD samples

	**Renewable Diesel - 1[a]**	**Renewable Diesel - D**	**Renewable Diesel - G**
0%	220	195	171
5%	270	262	225
10%	279	272	247
20%	284	287	270
30%	287	293	282
50%	290	298	287
70%	295	298	291
80%	294	296	292
90%	294	296	294
95%	294	297	296
100%	303	306	305

[a]Used for blending in this study.

Table S-6: Atmospheric (D86) distillation results for RD-1 and biodiesel blends

	**Renewable Diesel**	**RD- B20**	**RD-B40**	**RD-B60**	**RD B80**	**RD-B90**	**B100**
IBP	135.3	155.4	185.7	234.8	266.7	283.4	307.8
5%	262.9	266.0	272.6	283.6	302.9	315.8	330.3
10%	270.3	274.1	280.7	295.0	310.3	321.1	332.1
15%	275.4	280.6	287.2	298.3	313.3	323.7	333.0
20%	277.5	282.3	289.4	300.5	316.4	325.8	333.6
30%	280.6	286.2	294.1	306.4	321.6	329.1	334.4
40%	282.8	289.7	298.1	312.1	326.1	331.7	335.3
50%	284.8	292.6	302.8	318.2	329.7	333.8	336.1
60%	286.9	295.8	308.6	323.9	332.7	335.7	337.1
70%	289.2	300.2	315.5	329.6	335.2	337.5	338.2
80%	292.0	306.4	323.9	334.7	338.0	340.1	340.6
90%	295.9	317.8	333.9	340.2	344.9	347.3	347.8
95%	301.3	330.1	341.8	350.4	353.2		
FBP	311.3	340.8	346.5	350.6	353.2	349.7	347.8
Recovered, mL	97.1	98.2	98.2	98.0	98.0	96.6	96.5
Residue, mL	1.6	1.5	1.2	1.1	1.1	1.6	1.6
Loss, mL	1.3	0.3	0.6	0.9	0.9	1.8	1.9

Table S-7: Atmospheric (D86) distillation results for ULSD-A and biodiesel blends

	**ULSD-A**	**B60**	**B80**	**B90**
IBP	192.1	216.4	239.1	268.8
5%	223.2	254.2	283.3	307.7
10%	233.8	268.8	298.6	317.3
15%	238.9	277.2	307.6	322.8
20%	244.1	286.8	314.7	326.1
30%	252.7	303.9	324.2	330.8
40%	261.4	316.8	329.6	333.3
50%	270.8	325.5	332.5	335.1
60%	281.4	330.8	334.6	336.3
70%	294.1	334.5	336.5	337.9
80%	309.9	337.8	338.5	339.7
90%	333.9	342.5	344.5	346.4
95%	354.3	352.8	353.6	351.3
FBP	364.5	354.4	353.9	351.5
Recovered, mL	98.0	98.2	97.9	97.6
Residue, mL	1.1	1.0	1.0	1.4
Loss, mL	0.9	0.8	1.1	1.0

Table S-8: Vacuum (D1160) distillation results for RD-1 and biodiesel blends

	Renewable Diesel	RD- B20	RD-B40	RD-B60	RD B80	B100
IBP	168	176	183	195	198	245
5%	265	272	277	288	298	343
10%	278	283	293	297	319	346
20%	282	286	295	304	323	346
30%	286	290	298	318	333	348
40%	290	290	296	322	340	348
50%	292	285	295	329	344	349
60%	296	285	295	338	347	350
70%	298	287	296	345	349	350
80%	302	288	297	349	351	351
90%	306	288	298	351	352	352
95%	308	287	304	352	353	354
FBP	312	287	301	354	355	396

Table S-9: GC simulated (D2887 – D86 correlation) distillation results for RD-1 and biodiesel blends

	Renewable Diesel	RD- B20	RD-B40	RD-B60	RD B80
0%	220	221	230	239.0	257.5
5%	270	270	278	285.3	299.9
10%	279	281	286	296.2	310.0
20%	284	286	293	303.8	323.4
30%	287	291	297	318.8	333.2
50%	290	295	308	338.5	342.3
70%	295	304	332	340.3	341.6
80%	294	313	335	336.0	337.0
90%	294	326	333	334.1	335.1
95%	294	331	332	333.1	334.1
100%	303	335	336	336.3	336.4

Table S-10: GC Simulated (D2887 – D86 correlation) distillation results for ULSD-A and biodiesel blends

	ULSD-A	B60	B80	B90
0%	174	230.2	249.4	268.3
5%	200	255.6	283.3	332.8
10%	210	268.7	308.0	341.1
20%	226	293.9	334.2	353.7
30%	241	314.6	347.0	355.1
50%	267	340.1	344.2	344.8
70%	294	341.3	342.1	342.4
80%	310	336.9	337.5	337.7
90%	334	336.0	336.1	336.1
95%	355	336.2	335.5	335.3
100%	374	346.8	347.1	346.9

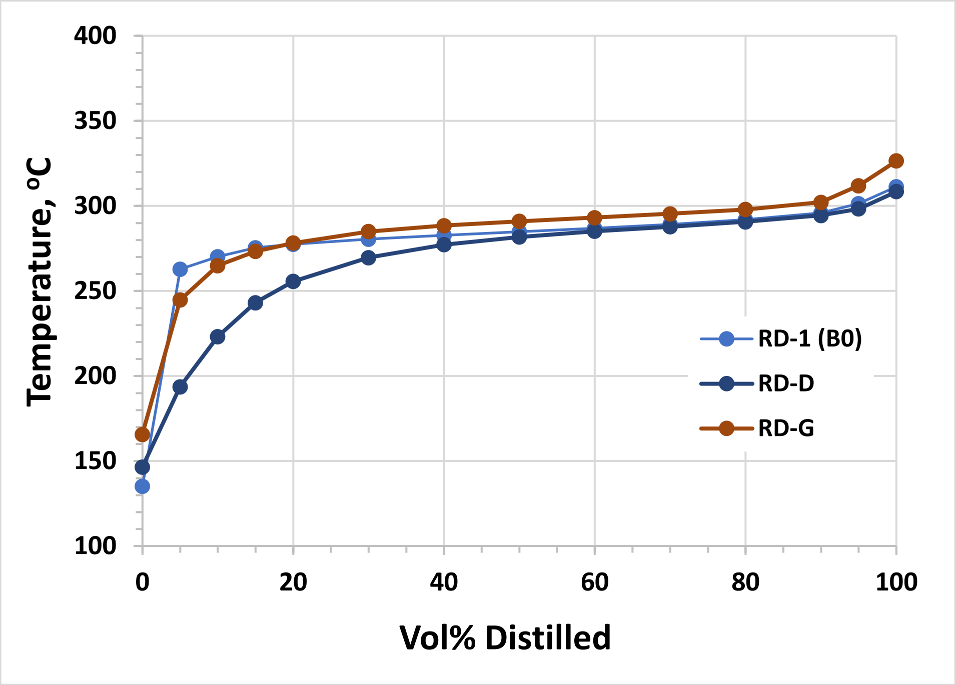

Figure S-1. D86 Distillation curves for RD-1, RD-D, and RD-G.

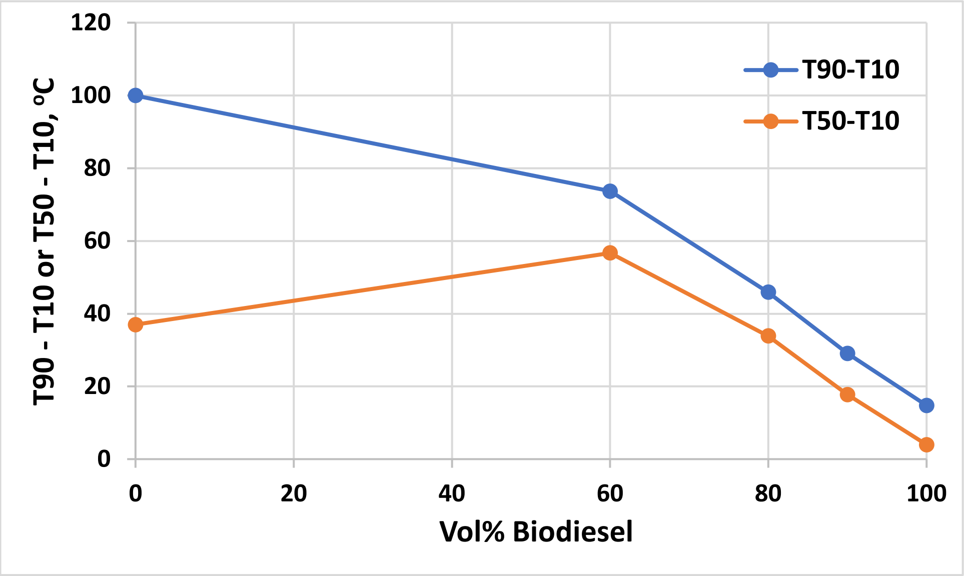

Figure S-2. T90-T10 and T50-T10 values for biodiesel blends in to ULSD-A.

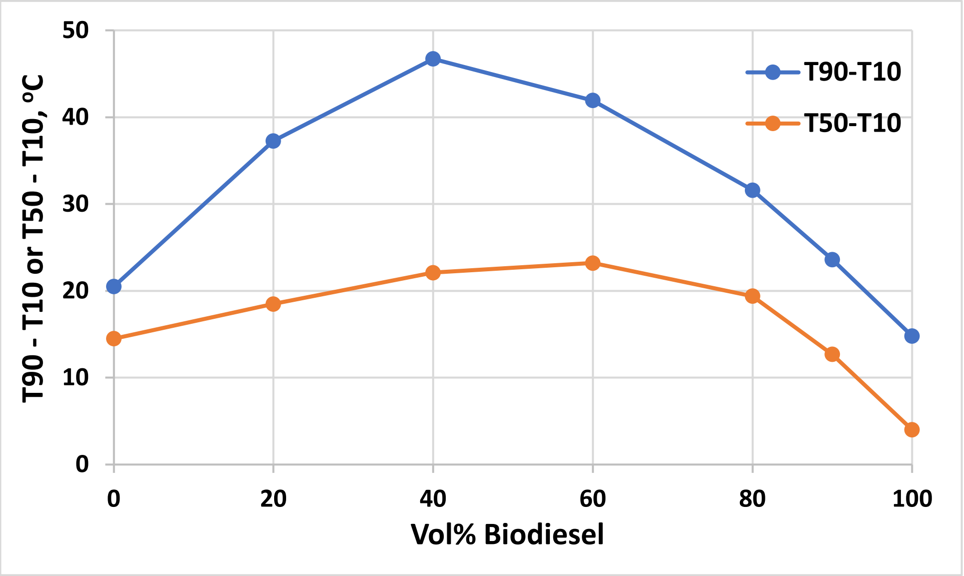

Figure S-3. T90-T10 and T50-T10 values for biodiesel blends into RD-1.
